# Factors associated with smoking in low-income persons with and without chronic illness

**DOI:** 10.18332/tid/138241

**Published:** 2021-07-15

**Authors:** Monique T. Cano, David L. Pennington, Sara Reyes, Blanca S. Pineda, Jazmin A. Llamas, Vyjeyanthi S. Periyakoil, Ricardo F. Muñoz

**Affiliations:** 1 Department of Psychiatry and Behavioral Sciences, University of California, San Francisco, San Francisco, United States; 2 Research Service, San Francisco VA Health Care System, San Francisco, United States; 3 Department of Psychology, University of Nebraska-Lincoln, Lincoln, United States; 4 Institute for International Internet Interventions for Health, Palo Alto University, Palo Alto, United States; 5 Stanford University School of Medicine, Stanford University, Palo Alto, United States

**Keywords:** mental health, tobacco-related disease, health disparities, health consequences

## Abstract

**INTRODUCTION:**

Tobacco disparities persist among low-income smokers who seek care from safety-net clinics. Many of these patients suffer from chronic illnesses (CILs) that are associated with and exacerbated by smoking. The objective of the current study was to examine the differences between safety-net patients with and without CILs in terms of nicotine dependence and related factors (such as depression, anxiety) and self-efficacy regarding ability to abstain from smoking.

**METHODS:**

Sixty-four low-income smokers who thought about or intended to quit smoking were recruited from the San Francisco Health Network (SFHN) and assessed for CILs, nicotine dependence, depression, anxiety, and smoking abstinence self-efficacy. Four one-way analyses of variance were used to examine the difference between those with and without CIL on the latter four variables.

**RESULTS:**

The CIL group had significantly higher anxiety (CIL: 8.0 ± 5.35; non-CIL: 4.44 ± 3.48; p=0.02) and tended to have higher nicotine dependence (CIL: 5.40 ± 2.58; non-CIL: 3.88 ± 2.28; p=0.04). In the CIL group, nicotine dependence was positively correlated with anxiety [r(62)=0.39; p<0.01] and negatively correlated with smoking abstinence self-efficacy [r(62)= -0.38; p<0.01]. Both depression (Spearman’s rho=0.39; p<0.01) and anxiety (Spearman’s rho=0.29; p<0.05) were associated with total number of CIL categories.

**CONCLUSIONS:**

Safety-net patients who smoke and suffer from CILs may be suffering from higher levels of anxiety and have less confidence in their ability to quit smoking. Incorporating mood management and developing interventions that increase a sense of self-efficacy for refraining from smoking may be necessary to help low-income smokers quit smoking.

## INTRODUCTION

Tobacco-related disparities and health disparities are strongly related; poverty, mental health, lower education levels, and unemployment are associated with high rates of smoking and in turn high rates of chronic illness (CIL)^1,2^. Many low-income smokers experience multiple medical, psychological, and social needs and often seek care from safety-net hospitals and clinics which are woefully under-resourced to address these concerns^3,4^. Smokers belonging to disadvantaged groups have higher rates of tobacco use, are less likely to make a quit attempt and are less likely to successfully quit, which significantly increases the risk for developing CILs^5^. Some factors that may contribute to continued tobacco use within this population include social determinants of health, minority status, higher levels of nicotine addiction, higher levels of stress, and co-occurring physical diseases and mental health diagnoses^1,2,6^. Higher smoking rates amongst lower social strata have persisted despite public health initiatives such as tobacco price increases, smoke-free policies, or robust tobacco control programs^7^.

Given the multiple medical, psychological, and social needs that low-income smokers face, the current study seeks to expand the literature on factors that underlie smoking in an underserved, low-income patient population. Exploring the underlying mechanisms that influence and are related to smoking behavior in low-income populations is crucial given their potential to inform the development of new interventions that are specific to the unique challenges of this social strata. In addition, low-income smokers who suffer from CILs are at significantly higher risk for poor health outcomes^8^ and may exhibit different underlying mechanisms associated with smoking behavior compared to smokers without CILs. In this study, we examined the differences between safety-net patients with and without CILs in terms of nicotine dependence and related factors (such as depression, anxiety) and self-efficacy regarding the ability to abstain from smoking.

Like smoking and social strata, mental disorders also independently increase the risk for developing CILs^2^. For instance, depression has been identified as an independent and prognostic factor for cardiovascular disease and cardiovascular incidents^9^. Thus, smokers diagnosed with mental disorders are disproportionately impacted by morbidity and mortality^10^. The disproportionality of this health disparity is significant given that over one-third of smokers suffer from serious psychological distress^11^. Smokers diagnosed with mental disorders smoke more cigarettes per day on average, are more dependent on nicotine, experience greater withdrawal symptoms during quit attempts, and have lower quit rates^12^. Moreover, most smokers who suffer from mental illness do not receive mental health treatment and have significantly lower quit rates in part because these individuals are more likely to be unemployed men with lower socioeconomic status (SES) and education levels, compared to smokers who are able to receive mental health treatment^13^.

The co-occurrence of smoking with other mental health disorders (e.g. anxiety, depression) is common and the incidence of one disorder occurring without co-occurring symptomatology is rare^14^. Notably, smoking prevalence rates increase with a greater number of mental disorders. Rates range from 18% for those with no mental disorder to 61% for those diagnosed with three or more mental health disorders^10,15^. However, most of the literature examining mental health disorders and smoking has generally focused on a singular clinical disorder^12^, resulting in a lack of knowledge regarding how to address smoking cessation in populations that present with multiple co-occurring mental health conditions^16^.

Prior research suggests that the presence of multiple health risks, e.g. smoking, low SES, depression, has a negative synergistic influence on morbidity and mortality^17^. Hence, studying underlying mechanisms that can influence behavior change across multiple domains and that have the potential for increased health benefits, is necessary. The facets of social cognitive theory (SCT) have been widely used to explain, identify, intervene and predict various factors that are associated with health behaviors^18^. One facet of SCT that has been shown to influence behavior is self-efficacy, which is the perceived confidence in one’s ability to perform specific behaviors in varying contexts^18^. For instance, self-efficacy has been identified as a predictor of smoking behavior and cessation^19^. Individuals who have a greater sense of self-efficacy are able to abstain from smoking in high-risk situations, e.g. when experiencing negative affectivity and when being faced with situational cues, such as being around other smokers, are more capable of bouncing back from a slip as opposed to proceeding to relapse^18,20^. The ability to modify self-efficacy has made it an appropriate outcome measure following smoking cessation interventions^19^. However, factors that influence self-efficacy, e.g. CIL status, nicotine dependence, depression and anxiety, and behavioral change may vary between persons with and without CIL.

According to SCT, smoking behaviors are influenced by the reciprocal determinism of cognitive, environmental, and behavioral factors^21^. These factors impact each other in a reciprocal manner meaning that causal changes could begin at each factor and the directionality of causation could involve many possibilities^21^. For example, smoking could lead to a CIL, which increases anxiety and depression, reduces self-efficacy for abstinence, increases likelihood of continuing to smoke, which then leads to more CILs and so on. However, this causal change could begin anywhere. For example, depression and anxiety could lead to smoking, which could then lead to CILs and so forth. Given that such a causal change has no necessary first cause in all persons, exploring factors that underly smoking behavior and utilizing that information to intervene where we can in the chain may be helpful in stopping the process.

Safety-net hospitals and clinics serve low-income patients with a myriad of medical, psychological, and social needs. Many of these patients smoke and suffer from CILs^3^. Although low-income smokers suffer disproportionately from health and tobacco related disparities, tobacco use among this patient population remains undertreated, understudied, and under-recognized as a problem^1,3,13^. To our knowledge, no prior research has explored differences in factors related to smoking – nicotine dependence, depression, anxiety, smoking abstinence self-efficacy – between low-income safety-net patients with and without CIL. Our primary hypothesis was that compared to those without CIL, participants with CIL would demonstrate greater nicotine dependence, higher depression and anxiety scores, and lower smoking-abstinence self-efficacy. In exploratory analyses, we also explored correlations between our outcome variables, such as nicotine dependence, depression, anxiety, smoking abstinence self-efficacy and: 1) the number of CILs in the two groups, and 2) each of six CIL categories (cardiovascular and circulatory disease, respiratory disease, endocrine disease, abdominal disease and obesity, chronic pain, and other disease).

## METHODS

### Participants

Participants were recruited from safety-net primary care clinics, within the San Francisco Health Network (SFHN) and other sites in the San Francisco Bay Area, that primarily serve low-income individuals such as community resource centers and community mental health clinics. Recruitment flyers were posted in waiting rooms and other locations with the approval of each of these sites. Staff at the various recruitment sites were also given flyers to inform prospective participants about the study. Eligible participants were low-income, as defined by the poverty threshold for San Francisco Bay Area residents^22^, adult (aged ≥18 years) tobacco smokers who have thought about or intended to quit smoking within 30 days.

### Measures

#### Demographic questionnaire

Participants reported their age, race, ethnicity, gender, sexual orientation, marital status, employment status, total household income, and years of education.

#### List of chronic illnesses

A self-report questionnaire asking for previous year presence or absence of 22 CILs were used to provide information on prevalence of CIL in the study sample. This questionnaire was modeled after the physical health self-report module in Atwoli et al.^23^. Similar to Atwoli et al.^23^, the 22 CILs were then aggregated by type into six disease categories: cardiovascular and circulatory disease (high blood pressure, high cholesterol, heart attack, stroke, heart disease), respiratory disease (asthma, seasonal allergies, other lung disease), endocrine disease (diabetes, thyroid, osteoporosis), abdominal disease and obesity (acid reflux, ulcer, obesity), chronic pain (arthritis, chronic pain, frequent headaches), and other disease (neurological disease, epilepsy, cancer, kidney disease, and other, to be specified by the participant).

#### Depression

The Patient Health Questionnaire 9 (PHQ-9) is a 9-item self-report questionnaire that assesses depression symptoms and severity^24^. The PHQ-9 assesses depressive symptomatology for the past two weeks. The questionnaire begins by asking ‘Over the past 2 weeks how often have you been bothered by ...’ followed by items such as, ‘Little interest of pleasure in doing things’ and ‘Feeling down, depressed, or hopeless’. Possible scores range from 0 to 27, with each of the 9 items scored from 0 (not at all) to 3 (nearly every day). Cut-off scores for the PHQ-9 in screening positive for a major depressive episode have ranged from 8–11. In this study, we used a cut-off score of ≥10 to categorize participants as screening positive for a major depressive episode^25^.

#### Anxiety

The Generalized Anxiety Disorder 7 (GAD-7) is a 7-item self-report questionnaire that assesses anxiety symptoms and severity^26^. The GAD-7 measures anxiety symptomatology for the past two weeks. The questionnaire begins by initially asking: ‘Over the past 2 weeks how often have you been bothered by ...’ followed by items such as ‘Trouble relaxing’ and ‘Worrying too much about different things’. Possible scores range from 0 to 21, with each of the 7 items scored from 0 (not at all) to 3 (nearly every day). In this study, we used a cut-off score of ≥8 to categorize a participant as screening positive for a diagnosable generalized anxiety disorder^25^.

#### Nicotine dependence

The Fagerström test of nicotine dependence (FTND) is a 6-item self-report measure that assesses nicotine dependence^27^. The measure includes items such as: ‘Do you smoke more frequently during the first hours after waking than during the rest of the day?’. Possible scores range from 0 to 10. A cut-off score of 6, the gold standard for detecting high nicotine dependence, was used in this study^27^.

#### Self-efficacy

The Smoking Abstinence Self-Efficacy Questionnaire (SASEQ) is a 6-item self-report questionnaire used to measure self-efficacy to refrain from smoking in specific contexts^28^. After reviewing the SASEQ with multiple researchers on the research team, it was determined that modifying questions for clarity and understanding, while not altering the content of the questions, was the best course of action before administering the measure as is. For example, one of the questions the SASEQ asks includes: ‘You feel agitated or tense. Are you confident that you will not smoke?’. This question was reworded as: ‘How confident are you that you will not smoke when you feel agitated or tense?’. The original response items for the SASEQ are on a 5-point Likert scale with responses ranging from ‘Certainly’ to ‘Certainly not’. The research team modified the responses for each question to include a 0–10 slider scale to provide more variability. Possible scores range from 0 to 60. The higher the score, the more self-efficacy one has to abstain from smoking. The original SASEQ has been shown to have high internal consistency with a Cronbach’s alpha of 0.8928. Internal consistency for the SASEQ that was used for this study yielded a Cronbach’s alpha of 0.86.

### Procedure

Participants were recruited from Zuckerberg San Francisco General Hospital (ZSFG), various clinics within the SFHN, and other surrounding areas in the San Francisco Bay Area that serve primarily low-income individuals. All participants provided informed consent, and study procedures were approved by the University of California, San Francisco and Palo Alto University. Data and analyses presented in the study were from self-report questionnaires that participants completed in-person at ZSFG on iPads.

### Statistical analysis

#### Power estimates

An a priori power analysis was performed for sample size estimation using G*Power (version 3.1.9.2)^29^. This study was designed with adequate power for a one-way analysis of variance of our primary hypothesis, namely participants with CIL will demonstrate greater nicotine dependence, higher depression and anxiety scores, and lower smoking-abstinence self-efficacy compared to those without CIL. These calculations indicate that a total sample size between 42 and 102 is required to detect a medium to large effect size (Cohen’s d between 0.5 and 0.8) between those with and without CIL. Given these power estimates, we expected our study sample of 64 low-income smokers to be appropriately powered to detect at least medium effect sizes. The standard alpha level (0.05) for main effects was adjusted for four multiple comparisons using a modified Bonferroni method^30^. This approach considers the mean correlation between outcome variables and the number of tests in the adjustment of alpha levels. We summed the intercorrelations among our four outcome measures and divided the result by the number of correlations used (average r=0.37). This correlation along with the 4 comparisons was used to derive an adjusted alpha level of p≤0.021 to determine statistical significance for the one-way analysis of variance. We also report any findings of our primary analysis that did not reach Bonferroni correction but were less than the standard statistical alpha of p<0.05 as trends towards significance. For exploratory correlational analyses a standard level p<0.05 was considered significant.

#### Descriptive and statistical model analyses

All statistical analyses were performed using the Statistical Package for Social Sciences (SPSS) version 26^31^. Demographic characteristics were compared using independent samples t-tests for continuous variables and the chi-squared test for categorical variables. For our primary hypotheses, four one-way analyses of variance were used to examine the difference between CIL group and those without CIL (our independent variables) on our four primary dependent variables: 1) smoking abstinence self-efficacy, 2) nicotine dependence, 3) depression, and 4) anxiety scores. Prior to analyses, dependent variable normality was confirmed. In exploratory analyses, we examined the associations between smoking abstinence self-efficacy, depression, anxiety, nicotine dependence, and the number of CIL categories (among the CIL group). Correlations with CIL disease categories were analyzed via Spearman correlations. All other associations were examined with Pearson correlations.

## RESULTS

### Participant characteristics

Descriptive statistics of participant characteristics are presented in Table 1; there were no statistically significant differences between the CIL group and the non-CIL group. The total sample consisted of 64 low-income smokers, 48 (75%) of which had at least one CIL. The CIL group included participants who indicated that they had the presence of at least one CIL. Characteristics consisted of 48 low-income smokers, 19 out of 48 were female (39.6%) and 1 (2.1%) of which identified as transgender; the age range for the CIL group was 31–70 years (46.64 ± 10.27). Over half of this group (31 out of 48; 64.6%) reported having completed high school or less, and 39 out of 48 (81.3%) reported having an annual income of less than $20000. Two-thirds (29 out of 48; 60.4%) of the CIL group reported having CILs in two or more of the CIL disease categories. The non-CIL group included participants who indicated that they did not have the presence of any CIL; characteristics consisted of 16 low-income smokers aged 24–59 years (41.69 ± 10.86), 2 (12.5%) of which were female. Over two-thirds (11 out of 16; 68.8%) of the non-CIL group reported completing high school or less, and 12 out of 16 (75%) reported making an annual income of less than $20000.

**Table 1:** Participant characteristics and group comparisons, SFHN 2018 (N=64)

*Characteristics*	*Non-CIL group (N=16)*	*CIL group (N=48)*
	*n (%)*	*n (%)*
**Gender**		
Female	2 (12.5)	19 (39.6)
Male	14 (87.5)	28 (58.3)
Transgender	0 (0.00)	1 (2.1)
**Age** (years) mean ± SD	41.69 ± 10.86	46.62 ± 10.07
**Education years,** mean ± SD	11.56 ± 2.53	12.43 ± 2.91
**Race**		
Black/African American	2 (12.5)	19 (39.6)
Asian	0 (0.0)	1 (2.1)
Mestizo	1 (6.3)	2 (4.2)
Native American/Alaskan Native	2 (12.5)	3 (6.3)
Native Hawaiian/Pacific Islander	1 (6.3)	1 (2.1)
White	6 (37.5)	14 (29.2)
Biracial	3 (18.8)	8 (16.7)
Unknown/Not reported	1 (6.3)	0 (0.0)
**Ethnicity**		
Latinx/Hispanic	6 (37.5)	10 (20.8)
Not Latinx/Hispanic	7 (43.8)	30 (62.5)
Unknown/Not reported	3 (18.8)	8 (16.7)
**Employment status**		
Full-time	4 (25)	2 (4.2)
Part-time	1 (6.3)	3 (6.3)
Retired	0 (0.00)	2 (4.2)
Student	1 (6.3)	7 (14.6)
Unemployed	10 (62.5)	34 (70.8)
**Household income** (US$)		
<20000	12 (75)	39 (81.3)
20000–34999	0 (0.0)	5 (19.4)
35000–49999	2 (12.5)	1 (2.1)
50000–74999	1 (6.3)	1 (2.1)
75000–99000	1 (6.3)	1 (2.1)
Unknown/Not reported	0 (0.0)	1 (2.1)

CIL: chronic illness. There were no statistically significant differences between CIL groups.

### Group difference in nicotine dependence, depression, anxiety, and self-efficacy by CIL status

A total of 15 participants (31.3%) in the CIL group reported PHQ-9 scores ≥10, thus screening positive for a major depressive episode compared to 3 (18.8%) in the non-CIL group (CIL: 7.69 ± 6.62; non-CIL: 5.13 ± 4.38; p=0.154). The CIL group had significantly higher anxiety scores compared to the non-CIL group (CIL: 8.0 ± 5.35; non-CIL: 4.44 ± 3.48; p=0.02) with a total of 23 participants in the CIL group (47.9%) having scores ≥8, thus screening positive for a generalized anxiety disorder compared to 3 (18.8%) in the non-CIL group. The CIL group also had higher nicotine dependence but did not reach significance given the significance level set using the Bonferroni adjustment (CIL: 5.40 ± 2.58; p=0.04); over half of the CIL group (52.1 %) reported FTND scores ≥6 indicating high nicotine dependence compared to the non-CIL group, in which 31.3% reported FTND scores ≥6. Results depicting group differences can be found in Table 2.

**Table 2:** Nicotine dependence, depression, anxiety, and self-efficacy group comparisons, SFHN 2018 (N=64)

	*Non-CIL group (N=16) Mean ± SD*	*CIL group (N=48) Mean ± SD*	*β*	*p*	*95% CI*
**Nicotine dependence**					
FTND total	3.88 ± 2.28	5.40 ± 2.58	-1.52	0.040**	(-2.97, -0.071)
**Depression**					
PHQ-9 total	5.13 ± 4.38	7.69 ± 6.62	-2.56	0.154	(-6.11, 0.989)
**Anxiety**					
GAD-7 total	4.44 ± 3.48	8.00 ± 5.59	-3.56	0.020*	(-6.54, -0.586)
**Self-efficacy**					
SASEQ total	25.13 ± 15.65	21.67 ± 12.74	3.46	0.378	(-4.33, 11.25)

CIL: chronic illness.

* p<0.02 Bonferroni corrected.

** p<0.05 indicating a trend towards significance.

FTND: Fagerström test of nicotine dependence. PHQ-9: Patient Health Questionnaire 9. GAD-7: Generalized Anxiety Disorder 7. SASEQ: Smoking Abstinence Self-Efficacy Questionnaire.

### Correlations between outcome variables and total number of CILs within non-CIL and CIL groups

The correlational matrices for both non-CIL and CIL groups are given in Table 3 and Table 4, respectively. In the non-CIL group, anxiety was positively correlated with depression [r(16)=0.61; p<0.05]. In the CIL group, nicotine dependence was positively correlated with anxiety [r(48)=0.39; p<0.01] and negatively correlated with smoking abstinence self-efficacy [r(48)= -0.38; p<0.01]. Among the CIL group, depression was also positively correlated with anxiety [r(48)=0.69; p<0.01] and the total number of CIL categories (Spearman’s rho=0.39; p<0.01). Finally, anxiety was positively associated with the total number of CIL categories (Spearman’s rho=0.29; p<0.05) among the CIL group.

**Table 3:** Correlations in non-chronic illness group, SFHN 2018 (N=16)

*Measure*	*1*	*2*	*3*
**Nicotine dependence**			
1 FTND			
**Depression**			
2 PHQ-9	0.44		
**Anxiety**			
3 GAD-7	0.31	0.61*	
**Smoking abstinence self-efficacy**			
4 SASEQ	0.17	-0.19	-0.11

* p<0.05.

FTND: Fagerström test of nicotine dependence. PHQ-9: Patient Health Questionnaire 9. GAD-7: Generalized Anxiety Disorder 7. SASEQ: Smoking Abstinence Self-Efficacy Questionnaire.

**Table 4:** Correlations in chronic illness group, SFHN 2018 (N=48)

*Measure*	*1*	*2*	*3*	*4*
**Nicotine dependence**				
1 FTND				
**Depression**				
2 PHQ-9	0.19			
**Anxiety**				
3 GAD-7	0.39**	0.69**		
**Smoking abstinence self-efficacy**				
4 SASEQ	-0.38**	-0.14	-0.16	
**Chronic illness**				
5 Number of CIL categories	-0.09	0.39**	0.29*	-0.01

* p<0.05.

** p<0.01.

FTND: Fagerström test of nicotine dependence. PHQ-9: Patient Health Questionnaire 9. GAD-7: Generalized Anxiety Disorder 7. SASEQ: Smoking Abstinence Self-Efficacy Questionnaire.

### Correlations between outcome variables and six chronic illness categories

The correlational matrices for each chronic illness category are given in Table 5. Anxiety and depression were positively correlated in each disease category: cardiovascular and circulatory [r(21)=0.50; p<0.05], respiratory [r(29)=0.64; p<0.01], abdominal and obesity [r(20)=0.62; p<0.01], chronic pain [r(22)=0.61; p<0.01], and other disease [r(10)=0.84; p<0.01] apart from endocrine disease. Nicotine dependence and smoking abstinence self-efficacy were negatively correlated in cardiovascular and circulatory disease [r(21)= -0.50; p<0.05] and chronic pain [r(22)= -0.54; p<0.05]. Nicotine dependence was positively correlated with anxiety in respiratory disease [r(29)=0.49; p<0.01], chronic pain [r(22)=0.58; p<0.01], and other disease [r(10)=0.67; p<0.05].

**Table 5:** Correlations among different chronic illness categories and outcome variables, SFHN 2018

*Chronic illness category*	*Measure*	*1*	*2*	*3*
Cardiovascular and circulatory disease (n=21)	1 Nicotine dependence			
	2 Depression	-0.50		
	3 Anxiety	0.31	0.50*	
	4 Smoking abstinence self-efficacy	-0.50*	0.01	0.04
Respiratory disease (n=29)	1 Nicotine dependence			
	2 Depression	0.78		
	3 Anxiety	0.49**	0.64**	
	4 Smoking abstinence self-efficacy	-0.25	0.05	-0.13
Endocrine disease (n=6)	1 Nicotine dependence			
	2 Depression	0.23		
	3 Anxiety	0.51	0.66	
	4 Smoking abstinence self-efficacy	-0.15	0.29	0.66
Abdominal disease and obesity (n=20)	1 Nicotine dependence			
	2 Depression	-0.04		
	3 Anxiety	0.34	0.62**	
	4 Smoking abstinence self-efficacy	-0.11	-0.21	-0.08
Chronic pain (n=22)	1 Nicotine dependence			
	2 Depression	0.80		
	3 Anxiety	0.58**	0.61**	
	4 Smoking abstinence self-efficacy	-0.51*	-0.03	-0.20
Other disease (n=10)	1 Nicotine dependence			
	2 Depression	0.35		
	3 Anxiety	0.67*	0.84**	
	4 Smoking abstinence self-efficacy	-0.54	-0.22	-0.43

* p<0.05.

** p<0.01.

## DISCUSSION

This study examined the differences between smokers with and without CILs in terms of nicotine dependence, depression, anxiety, and smoking abstinence self-efficacy. The goal of the study was to gain a greater understanding of the factors that influence smoking behavior in a population that disproportionately suffers from morbidity and mortality. We found that individuals who have CIL exhibited higher anxiety scores and tended to be more addicted to nicotine than those without CIL. Anxiety was associated with higher nicotine dependence and depression among the CIL group as well as those with respiratory disease, chronic pain, and other disease; depression and anxiety were both positively associated with the number of CIL categories and each disease category apart from endocrine disease. Nicotine dependence was also negatively associated with smoking abstinence self-efficacy among the CIL group, in those with cardiovascular and circulatory disease and in those with chronic pain. Among smokers in the non-CIL group, anxiety was positively correlated with depression.

The social determinants of health that contribute to health disparities are linked to tobacco-related disparities; smokers of disadvantaged groups have higher levels of nicotine dependence and mental health diagnoses both of which markedly increase the risk for developing CILs^5,6^. We found significant differences between groups on anxiety and notable differences on nicotine dependence. In this study, smokers with CIL reported higher levels of anxiety and tended to be more addicted to nicotine compared to smokers without CIL. These results are in line with previous research which has found that patients with CIL tend to experience higher levels of anxiety and depression than those without^32^. One possible explanation for higher levels of anxiety is that smokers with CIL may be experiencing greater anxiety because of their chronic conditions, as individuals who cope with CIL commonly report fears and stressors associated with illness symptoms recurring and/or worsening^33^. Similarly, we found that both depression and anxiety were related to number of CIL categories and although there were no significant differences between CIL and non-CIL groups in terms of depression and anxiety, there were more smokers in the CIL group who screened positive for a major depressive episode and generalized anxiety disorder. Thus, patients who suffer from one or more CIL are more likely to experience anxiety and depression. This finding is consistent with previous research, indicating that coping with more than one CIL is associated with an increase in the occurrence of depression and anxiety^34,35^.

Anxiety and depression are common co-occurring mental health diagnoses in smokers and those with CIL^36^. Although smoking may exacerbate and worsen symptoms of CILs, individuals with anxiety and depression may be smoking more to cope with their mental health symptoms which in turn increases their nicotine dependence. Nicotine’s psychoactive effects, which are associated with mood modulation and the reduction of stress, make it increasingly difficult for individuals to quit smoking despite it being deleterious to their health^37^. Consistent with previous studies^38^, this study showed that anxiety is linked to nicotine dependence. Specifically, we found that smokers in the CIL group with higher nicotine dependence (FTND scores) had higher anxiety symptoms (GAD-7 scores); likewise, we found this association among those who indicated having respiratory disease, chronic pain, and other forms of disease. It may be important for clinicians to assess for anxiety in smokers with CIL, especially in patients who suffer from respiratory illnesses such as chronic obstructive pulmonary disease (COPD) and chronic pain. When providing smoking cessation treatment, it is important to remember that anxiety may worsen in patients who are attempting to quit smoking and make provisions to provide treatment for anxiety as well as appropriate psychosocial support to empower successful quitting of smoking. It is also important to routinely screen patients with CIL for anxiety and depression during medical encounters. Future studies are needed to evaluate whether routine assessment and monitoring anxiety symptoms in CIL patients: a) will improve health outcomes, and b) whether specific anxiety-related interventions may improve smoking cessation in low-income smokers who have CIL.

Among smokers with CIL, nicotine dependence and smoking-abstinence self-efficacy were negatively correlated; correspondingly, this association was also found in those who indicated having cardiovascular and circulatory disease and chronic pain. This suggests that within this sample, individuals who are more dependent on nicotine are also less confident in their ability to abstain from smoking in high-risk situations, e.g. when faced with situational cues or when experiencing negative affectivity, because of the cravings these situations induce, the desire to relieve withdrawal symptoms, and the need to regulate mood^18,20,37^. These findings are in line with previous research indicating that individuals with a lower sense of self-efficacy find it more difficult to resist smoking in high-risk situations and are more likely to relapse^18,20^. However, in this sample of low-income smokers, this relationship only existed among those who have CIL. This result reveals that smokers who have a CIL may have a harder time quitting smoking than those who do not. Understanding how self-efficacy influences smokers who present with multiple health risks is important because self-efficacy in one behavioral domain (e.g. smoking cessation) may impact, e.g. increase or decrease, self-efficacy in other behavioral domains that influence morbidity and mortality; for example, success in quitting smoking may also increase one’s self-efficacy to improve other modifiable health risks such as the behaviors associated with depression^17^ which in turn may increase CIL self-management and reduce the susceptibility and severity of CILs.

Although previous studies have highlighted the need for clinicians to promote a sense of confidence in a smoker’s abilities to abstain from smoking^39^, few treatments are designed to improve self-efficacy despite many studies stressing the need for these interventions^20^. However, this clinical implication must be adapted and applied appropriately to low-income populations who suffer from multiple co-occurring diseases/disorders. Given the complexities involved in treating low-income smokers who also have CILs and may be suffering from mental disorders, future research is needed in developing intensive interdisciplinary smoking cessation interventions with a focus on CIL symptom self-management, coping strategies for managing distress in triggering situations, and confidence-building. While many hospital systems provide smoking cessation resources, a continued emphasis must be placed on offering low-cost or free resources (such as free smoking cessation apps), nicotine replacement therapies (NRTs), individual and/or group psychotherapy, and pharmacologic aids. Understanding that this population requires more support for quitting given their socioeconomic status and multiple morbidities, hospital systems and community clinics should obtain the resources necessary to support these patients and implement quality improvement (QI) initiatives to determine whether providing these resources improves abstinence rates and chronic disease management.

### Limitations

This study has some limitations. First, the cross-sectional and self-report nature of the research design does not allow for causal interpretations of the results. Although number of CILs were related to both anxiety and depression in smokers, the bidirectionality of these relationships limits our ability to interpret their etiological nature and thus, limits our ability to provide preventative clinical implications. Similarly, although we were able to garner meaningful insights into factors that are associated with smoking cessation in a low-income population, we were not able to measure changes through time. Second, an inclusion criterion consisted of whether participants had thought about or intended to quit smoking in 30 days; this criterion may have captured smokers at differing levels of motivation which could have influenced self-efficacy outcomes. Third, the study had a relatively small sample size which affected statistical power and limited our ability to run more robust analyses. Fourth, this sample is limited to low-income smokers who use a public sector healthcare system. It is possible that this sample may represent only treatment seeking low-income smokers. Individuals who are not involved in treatment or connected to care may have differing levels of motivation to quit as well as differing levels of mental health and self-efficacy. For example, low-income smokers who are not treatment seeking may have little motivation to quit despite having a CIL diagnosis. Fifth, while the study’s outcomes may be associated with a lower likelihood of smoking cessation, this study did not measure smoking cessation directly. Within this selected sample of low-income smokers, there may have been other factors that contribute to smoking cessation such as previous quit attempts that were not measured. Sixth, given the voluntary nature of this study, results presented cannot be generalized to those who chose not to participate. Similarly, the data presented here are from a low-income population in an urban area of the United States, the results may not generalize to other low-income populations who reside in rural areas or areas outside the United States. Nevertheless, this research contributes to a body of literature in a low-income population that has historically been understudied and found that low-income smokers with CIL may need more intensive cessation treatments since they exhibit characteristics that may make it more difficult to quit smoking^5,13^.

Future research in this area is warranted. Low-income smokers with CILs need to receive adequate smoking cessation treatments. Given the multiple co-occurring diseases/disorders that low-income smokers experience, comprehensive interdisciplinary treatments are needed. Additionally, future research should include a qualitative component to gain a better understanding of the internal and external challenges that low-income smokers face when they decide to quit smoking.

Although routine screening occurs in some large healthcare systems^3^, the following recommendations should be implemented to reduce the number of low-income patients with CILs that are tobacco related: 1) routine screening for smoking, depression, and anxiety; 2) providing smoking cessation interventions that include mood management interventions; and 3) developing interventions that increase a sense of self-efficacy for refraining from smoking.

## CONCLUSIONS

Research consistently shows that low-income smokers suffer disproportionately from tobacco-related disparities and have a higher risk for morbidity and mortality^1–4^. Therefore, it is important to gain a greater understanding of the factors that underlie smoking in marginalized populations. In this study, relative to smokers who do not have chronic illness (CIL), smokers with CIL reported higher levels of anxiety and tended to be more addicted to nicotine; among those with CIL, nicotine dependence and self-efficacy were related. Assessing for anxiety and depression and incorporating mood management interventions with self-efficacy components may be necessary to help low-income smokers quit.

## Data Availability

The data supporting this research is available from the authors on reasonable request.
